# The efficacy analysis of immunotherapy rechallenge after progression from first-line chemo-immunotherapy in advanced non-small cell lung cancer

**DOI:** 10.1186/s12865-026-00800-4

**Published:** 2026-01-27

**Authors:** Wenhao Shi, Rui Kong, Jin Xiong, Yuan Peng, Zhenzhou Yang, Yusheng Huang

**Affiliations:** 1 Department of Cancer Center, The Second Affiliated Hospital, Chongqing Medical University, Chongqing, 400010 China; 2 Department of Cancer Center, The Third Affiliated Hospital, Chongqing Medical University, Chongqing, 400016 China; 3 Chongqing Key Laboratory of Immunotherapy, Chongqing, 400037 China; 4 Guchengtai Community Health Center of Chengxi District, Xining, 810000 China; 5 Department of Cancer Center, The Second Affiliated Hospital of Chongqing Medical University, No. 288 Tianwen Road, Nan’an District, Chongqing, 400072 China

**Keywords:** Non-small cell lung cancer (NSCLC), Immunotherapy rechallenge, Prior immunotherapy response, Anti-angiogenesis therapy

## Abstract

**Introduction:**

Combining immune checkpoint inhibitors (ICI) with chemotherapy has been established as the standard first-line (1 L) treatment for advanced non-small cell lung cancer (NSCLC). However, the optimal second-line (2 L) treatment following 1 L chemo-immunotherapy remained controversial.

**Methods:**

Patients with advanced NSCLC who progressed following 1 L chemo-immunotherapy and continued to receive ICI as 2 L therapy were enrolled in the study. These patients were divided into two groups according to the therapeutic modality: chemo-immunotherapy plus anti-angiogenesis therapy group (CIA group) and chemo-immunotherapy group (CI group). Baseline characteristics were balanced using inverse probability of treatment weighting (IPTW) to minimize selection bias before comparative analyses. The efficacy of immunotherapy rechallenge combination therapy was evaluated based on overall survival (OS) and progression-free survival (PFS). Cox proportional hazards regression analyses were applied to determine predictive factors independently associated with survival outcomes.

**Results:**

A total of 101 patients were enrolled in this study, 45 patients were enrolled in CIA group, and those who didn’t receive anti-angiogenesis drugs were defined as CI group (*n* = 56). Overall, Immunotherapy rechallenge reached a median OS of 19.47 months and median PFS of 7.10 months. The CIA group showed significantly longer PFS than the CI group (11.49 vs. 6.06 months, *p* = 0.03). Similar results in PFS were observed in the study cohorts balanced with IPTW (11.49 vs. 6.89, *p =* 0.01). Multivariate analyses revealed that prior immunotherapy response and smoking status could be independent prognostic factors for PFS.

**Conclusions:**

Immunotherapy rechallenge could bring survival benefits following progression under chemo-immunotherapy, especially those who responded to prior immunotherapy. Additionally, the use of anti-angiogenesis drugs could significantly improve the clinical response of immunotherapy rechallenge.

**Supplementary Information:**

The online version contains supplementary material available at 10.1186/s12865-026-00800-4.

## Introduction

Non-small cell lung cancer (NSCLC) accounts for approximately 85% of all lung cancer cases, which is the leading cause of cancer deaths worldwide [1]. As we embark on the immunotherapy epoch, immune checkpoint inhibitors (ICI) have revolutionized the treatment landscape and significantly boosted survival outcomes for patients with lung cancer. Numerous clinical studies, including KEYNOTE 407, have demonstrated that the combination of chemotherapy and immunotherapy as a first-line (1 L) treatment exhibits superior effectiveness, achieving a median overall survival (OS) of 12 months and an estimated 5-year survival rate of 2% for advanced NSCLC. However, progression after 1 L immunotherapy occurs in the majority of patients shortly thereafter [2]. Despite recent therapeutic advancements, a notable proportion of patients with advanced NSCLC remain unresponsive to immunotherapy and continue to have a poor prognosis, thus requiring standard second-line (2 L) therapy strategy. Docetaxel is widely used as the preferred subsequent systemic therapy, but it has limited survival benefits, with a reported OS of 6.0-9.1 months [3–5]. Currently, immunotherapy rechallenge after developing drug resistance has gained popularity. Initial melanoma and advanced renal cell carcinoma trials demonstrated that immunotherapy rechallenge can effectively regain disease control [6–8]. Additionally, a prior or concomitant radiotherapy, chemotherapy or anti-angiogenesis therapy were proposed as promising strategies to improve the efficacy of primary and secondary immunotherapy [9–13]. Therefore, for patients with advanced NSCLC who showed disease progression after treating with ICIs, the continuation of immunotherapy combined with traditional antitumor treatment regimens is still in controversial hotspots.

Anti-angiogenesis converts the tumor immune environment from immuno-suppressive to immuno-supportive status [14]. Therefore, immunotherapy plus anti-angiogenesis therapy is widely used as an optimal strategy that achieves promising clinical outcomes for solid tumors, such as renal cancer, hepatocellular carcinoma, and non-squamous NSCLC [15–17]. In the Impower-150 study, the combination of chemo-immunotherapy and anti-angiogenesis has demonstrated beneficial survival outcomes as primary first-line treatments for advanced NSCLC patients [18]. Given these survival advantages, it is also reasonable to consider employing this combination strategy in second-line therapy to further enhance therapeutic efficacy.

We performed this retrospective analysis to explore immunotherapy continuation therapy’s efficacy and identify the optimal combination therapy strategy in second-line therapy for advanced NSCLC after progression following 1 L chemo-immunotherapy. Finally, we hope to provide insightful guidance and recommendations in developing 2 L treatment strategies for NSCLC patients who showed disease progression after 1 L chemo-immunotherapy in the future.

## Materials and methods

### Patients

We retrospectively reviewed the medical records of 101 patients with advanced NSCLC whose treatment was continued with anti-PD-1 to anti-PD-L1 antibodies as ICI rechallenge between August 2021 and November 2023. Inclusion criteria comprised: (1) confirmation of NSCLC through histology or cytology (2) stage IIIC/IV (3) confirmed disease progression following 1 L chemo-ICI treatment (ICI involving anti-PD-1 or anti-PD-L1) and continued to receive ICI as 2 L treatment (5) at least one lesion quantifiable by the Response Evaluation Criteria for Solid Tumors (RECIST 1.1) guidelines, version 1.1. Individuals treated with targeted therapies (TKI) or experimental medication were not considered eligible for inclusion. The required data were extracted from the electronic medical records system using a standardized data collection form.

### Endpoint

The primary endpoint of this study was to assess the efficacy of immunotherapy rechallenge combination therapy through the OS, PFS, ORR, and DCR. OS: the time between initiation of the post-PD treatment and death from any cause; PFS: the duration from the initiation of the post-PD treatment to disease progression or death from any cause, whichever came first; Objective Response Rate (ORR): the percentage of patients who experience either a complete response (CR) or a partial response (PR) following therapy; Disease Control Rate (DCR): encompasses the ORR, integrating it with instances of stable disease (SD) as determined by clinical practitioners. A minimum period of three weeks was necessary to verify Complete Response (CR) and Partial Response (PR). In contrast, the evaluation for Stable Disease (SD) required at least a single radiological examination following six weeks from the start of the treatment. Moreover, Cox logistic regression analyses were used to explore prognostic effects on the survival outcome of advanced NSCLC patients.

### Treatments

The patients were divided into chemo-immunotherapy plus anti-angiogenesis (CIA) group and chemo-immunotherapy (CI) group based on their treatment modality. The PD-1/PD-L1 inhibitors included sintilimab, pembrolizumab, nivolumab, camrelizumab and toripalimab. The chemotherapy regimens involved liposome paclitaxel, nab-paclitaxel, docetaxel, pemetrexed, and others. The anti-angiogenic drugs included bevacizumab, anlotinib, and endostatin. More detailed plans are available in Table S1. The tumor response to initial immunotherapy treatment reflects the patient’s sensitivity to immunotherapy. For patients with 1 L-PFS ≥ 6 months after receiving immunotherapy, we define them as responders. If disease progression occurs within 6 months, defined as 1 L-PFS less than 6 months, these patients are classified as resistant.

### Statistical analysis

This study presented median values and interquartile range for continuous variables and frequencies with percentages for categorical variables. The means and proportions were compared by employing the Student’s t-test and the chi-square test (or Fisher’s exact test when appropriate). To mitigate selection bias and equalize baseline characteristics across the two groups receiving combination therapies, the inverse probability of treatment weighting (IPTW) approach was applied. The standard mean difference (SMD) was computed to assess the equilibrium between the groups, with values of ≤ 0.1 denoting optimal balance, and those ≤ 0.2 indicated an acceptable balance. Survival curves for each treatment group were plotted using the Kaplan-Meier method to estimate the median OS and median PFS, and comparisons were made using the log-rank test. A Cox proportional hazards model performed multivariate survival analysis to evaluate the independent prognostic factors associated with improved survival. The log-rank test estimated the difference in survival curves between the two groups. P-values less than 0.05 were deemed to indicate statistical significance, and all tests were conducted on a two-sided. Statistical analyses were executed using R software (version 3.6.1; http://www.R-project.org), developed by the R Foundation for Statistical Computing, based in China.

## Results

### Baseline characteristics

A total of 101 patients were enrolled in the analysis. Forty-five patients (44.6%) received chemo-immunotherapy plus anti-angiogenesis combination therapy, and 56 (55.4%) received chemo-immunotherapy. The average age across the cohort was 60 years, with females comprising 16.8% of the enrolled patients and 45.5% diagnosed with squamous. Before commencing second-line (2 L) therapy, the performance status (PS) was either 0 or 1 for 99 patients, representing 98.0% of the cohort. The prevalence of brain metastasis was observed at 15.6% in the CIA group compared to 14.3% in the CI group. The median duration of follow-up was 11.4 months (range 2.1–24.7 months) for the enrolled patients. Moreover, there were a total of 20 cases (19.8%) have experienced genetic mutations: 6 patients (5.9%) with tumors harboring KRAS mutations, 6 patients (5.9%) with tumors harboring TP53 mutations, three patients (2.9%) with tumors harboring EGFR mutations. Among the 101 patients, 31 (30.7%) patients had a tumor considered resistant to 1 L chemo-immunotherapy (1 L-PFS **<** 6 months), and 70 (69.3%) patients were responders (1 L-PFS ≥ 6 months). After applying IPTW, the balance between the two treatment groups improved. More information is available in Table 1.

**Table 1:** Baseline characteristics of patients receiving anti-angiogenesis versus non-anti-angiogenesis in unadjusted and IPTW-adjusted study populations

	Observed cohort before IPTW					Banlanced cohort after IPTW			
**Characteristic**		CIA group	CI group	*p*	SMD	CIA group	CI group	*p*	SMD
Age	60 [52.2–62.9]			0.172	0.323			0.928	0.021
≥ 65	39 (38.6%)	11 (24.4%)	22 (39.3%)			31.3 (31.3)	31.6 (32.3)		
< 65	62 (61.4%)	34 (75.6%)	34 (60.7%)			68.6 (68.7)	66.3 (67.7)		
Sex				0.303	0.259			0.785	0.062
Male	84 (83.2%)	35 (77.8%)	49 (87.5%)			80.3 (80.4)	81.0 (82.8)		
Female	17 (16.8%)	10 (22.2%)	7 (12.5%)			19.6 (19.6)	16.9 (17.2)		
Performance status				0.17	0.378			0.106	0.249
0–1	99 (98.0%)	42 (93.3%)	56 (100.0%)			96.9 (97.0)	97.9 (100.0)		
2	2 (2.0%)	3 (6.7%)	2 (0.0%)			3.0 (3.0)	0.0 (0.0)		
Smoking				0.443	0.196			0.920	0.021
Yes	64 (63.4%)	28 (62.2%)	40 (71.4%)			69.0 (69.0)	68.5 (70.0)		
No	37 (36.6%)	17 (37.8%)	16 (28.6%)			30.9 (31.0)	29.4 (30.0)		
Pathology				0.588	0.369			0.835	0.046
Squamous	46 (45.5%)	16 (35.6%)	30 (53.6%)			46.7 (46.7)	43.5 (44.4)		
Non-squamous	55 (54.5%)	29 (64.4%)	26 (46.4%)			53.2 (53.3)	54.4 (55.6)		
Mutation				0.425	0.209			0.844	0.043
Yes	20 (19.8%)	11 (24.4%)	9 (16.1%)			20.7 (20.7)	18.6 (19.0)		
No	81 (80.2%)	34 (75.6%)	47 (83.9%)			79.2 (79.3)	79.3 (81.0)		
Prior immunotherapy response				1.000	0.016			0.824	0.051
Resistant (1 L PFS < 6 months)	31 (30.1%)	14 (31.1%)	17 (30.4%)			37.0 (37.0)	33.8 (34.6)		
Responder (1 L PFS ≥ 6 months)	70 (69.9%)	31 (68.9%)	39 (69.6%)			62.9 (63.0)	64.1 (65.4)		
Second-line radiotherapy				0.383	0.217			0.843	0.043
Yes	35 (34.7%)	20 (44.4%)	15 (26.8%)			40.3 (40.3)	37.4 (38.2)		
No	66 (65.3%)	25 (55.6%)	41 (73.2%)			59.6 (59.7)	60.5 (61.8)		
Primary brain metastasis				1.00	0.036			0.997	0.001
Yes	15 (14.9%)	7 (15.6%)	8 (14.3%)			12.8 (12.8)	12.5 (12.8)		
No	86 (85.1%)	38 (84.4%)	48 (85.7%)			87.1 (87.2)	85.4 (87.2)		
Previous radiotherapy				0.10	0.375			0.978	0.006
Yes	35 (34.7%)	20 (44.4%)	15 (26.8%)			33.2 (33.2)	32.8 (33.5)		
No	66 (65.3%)	25 (55.6%)	41 (73.2%)			66.7 (66.8)	65.1 (66.5)		

### Response

In the cohort of 101 patients, the 2 L-ORR was found to be 24.4% in the group receiving chemo-immunotherapy in combination with anti-angiogenesis therapy, compared to 12.5% in the group treated with chemo-immunotherapy alone (*p* = 0.194). The 2 L-DCR was 62.2% in the CIA group and 42.9% in the CI group (*p* = 0.083). These differences in response rates between the two groups did not reach statistical significance, as detailed in Table 2.

**Table 2:** Overall response

	CIA Group(*n* = 45)	CI Group(*n* = 56)
Objective response rate, n (%)	11(24.40%)	7(12.50%)
*P* value	0.194	
Disease control rate, n (%)	28(62.20%)	24(42.90%)
*P* value	0.083	
Best overall response, n (%)		
CR	0	0
PR	11 (24.4%)	7 (12.5%)
SD	17 (37.8%)	17 (30.4%)
PD	17 (37.8%)	32 (57.1%)

*CR* complete response, *PR* partial response, *SD* stable disease, *PD* progressive disease

### Efficacy

**Fig. 1:**
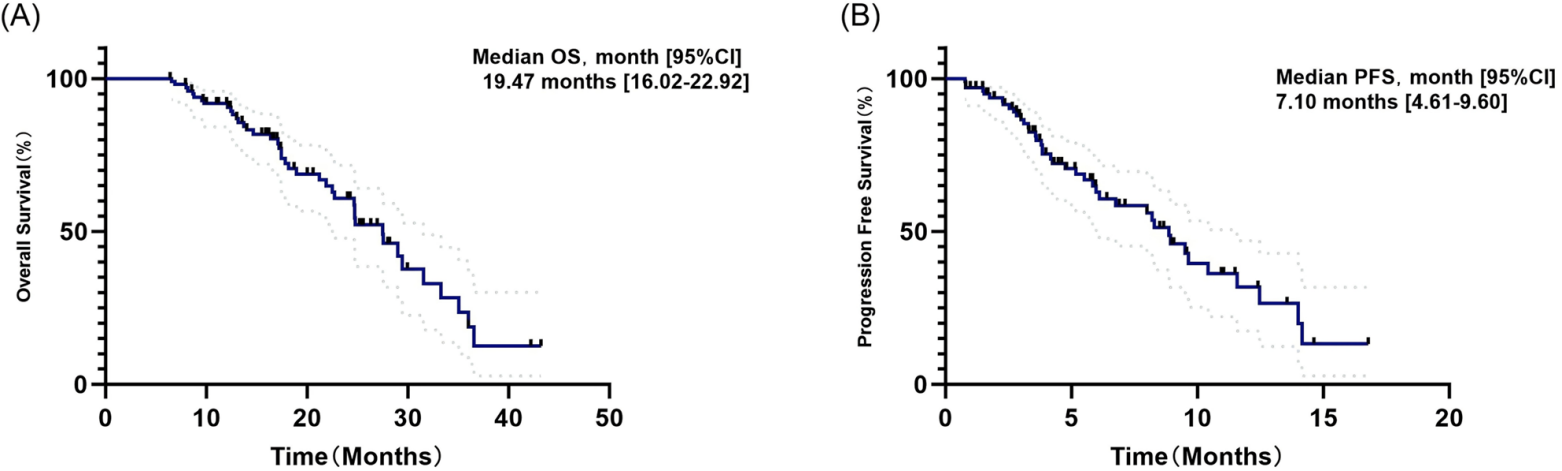
Kaplan-Meier analysis of OS and PFS in patients with advanced NSCLC receiving second-line immunotherapy rechallenge (**A**) Kaplan-Meier analysis OS. **B** Kaplan-Meier analysis of PFS. NSCLC, non-small-cell lung cancer; immunotherapy rechallenge, immunotherapy continuation after progression from first-line chemo-immunotherapy; OS, overall survival; PFS, progression-free survival

**Fig. 2:**
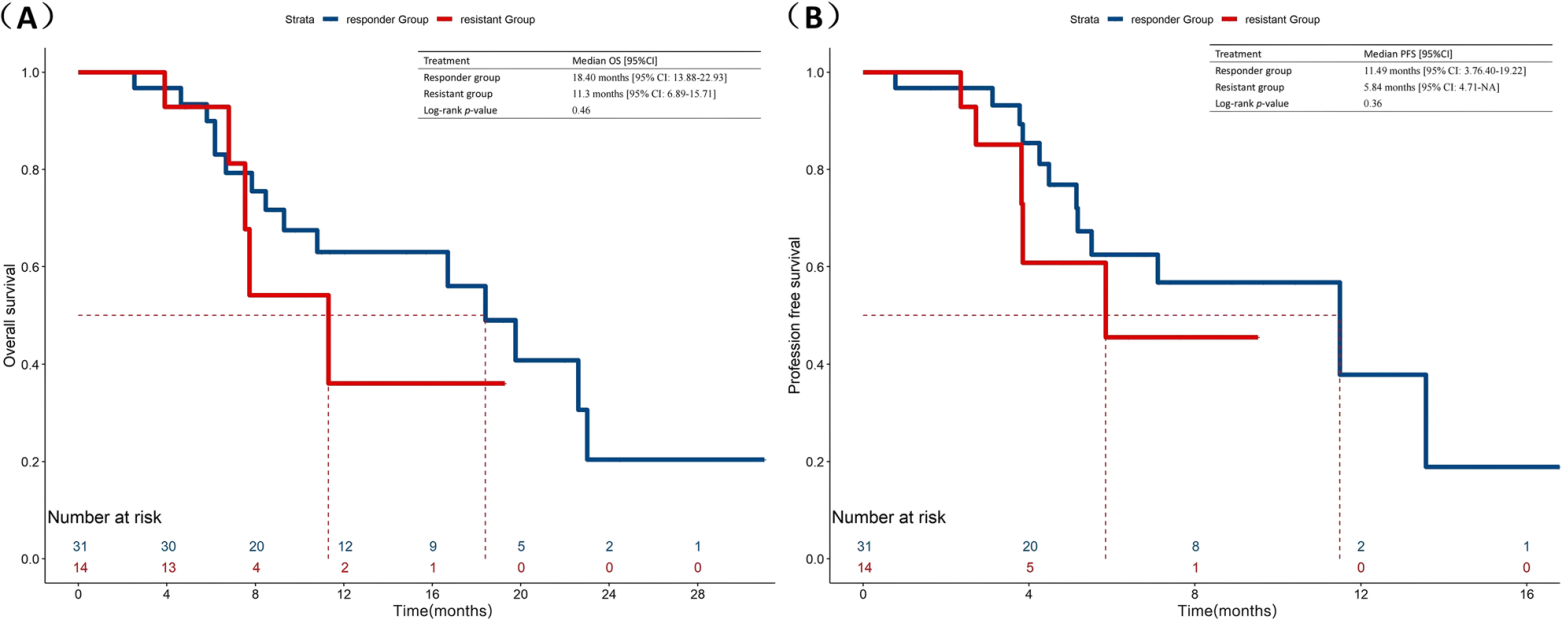
Comparison of OS and PFS in patients with advanced NSCLC receiving second-line immunotherapy rechallenge between chemo-immunotherapy plus anti-angiogenesis therapy and chemo-immunotherapy. **A** Comparison of OS. **B** Comparison of PFS

**Fig. 3:**
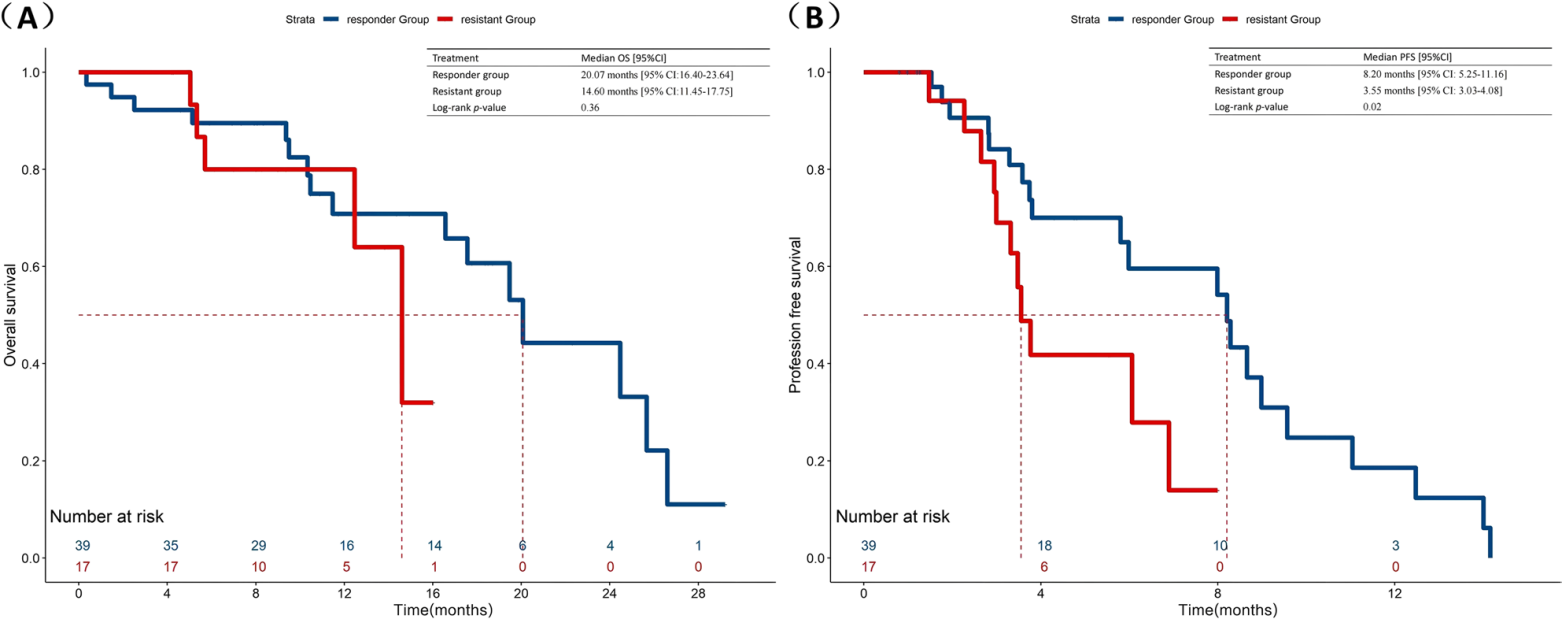
Comparison of OS and PFS in patients with advanced NSCLC receiving second-line immunotherapy rechallenge between chemo-immunotherapy plus anti-angiogenesis therapy and chemo-immunotherapy after IPTW. **A** Inverse probability of treatment weighting-adjusted Kaplan-Meier curves of OS for patients in CIA Group versus CI Group. **B** Inverse probability of treatment weighting-adjusted Kaplan-Meier curves of PFS for patients in CIA Group versus CI Group. IPTW, Inverse probability of treatment weighting. CIA Group, chemo-immunotherapy plus anti-angiogenesis therapy. CI Group, chemo-immunotherapy

The overall population median OS was 19.47 months (95%CI:16.02–22.92) (Fig. 1A). In the CIA group, they had a median OS of 18.40 months (95% CI: 9.70-27.11), compared with 19.47 months (95% CI: 16.09–22.84) in the CI group (*p* = 0.36) (Fig. 2A). In terms of short-term survival outcomes, immunotherapy rechallenge attained a median PFS of 7.10 months (95% CI: 4.61–9.60) (Fig. 1B). The median PFS was 11.49 months (95% CI: 3.63–19.35) for the patients receiving anti-angiogenesis drugs on the basis of chemo-immunotherapy and 6.06 months (95% CI:3.70–8.42) for those only receiving chemo-immunotherapy (*p* = 0.03) (Fig. 2B). After IPTW analysis, according to the regimens administered, the median PFS was still more prolonged in the CIA group than in the CI group (11.49 vs. 6.89, *p =* 0.01), although no significant difference was observed in OS (16.70 vs. 19.47, *p* = 0.06) (Fig. 3).

The clinical characteristics of the patients were evaluated to determine their prognostic value for PFS. Univariate analysis indicated that variables including prior immunotherapy response and smoking status were associated with survival. Multivariate analyses continued to indicate that prior immunotherapy response (*p* = 0.035) and smoking status (*p* = 0.004) still were significant favorable prognostic factors (Table 3).

**Table 3:** Univariate and multivariate analysis of factors associated with PFS

Factors	Univariate analysis		Multivariate analysis	
	HR (95% CI)	*P* value	HR (95% CI)	*P* value
Age (≥ 65 vs. <65 years)	0.87–2.87	0.13	-	-
Sex (female vs. male)	0.60–2.82	0.50	-	-
Smoking status (yes vs. no)	0.21–0.72	0.003	0.22–0.75	0.004
Pathology (Adenocarcinoma vs. Squamous)	0.54–1.70	0.89	-	-
Mutation (yes vs. no)	0.47–1.84	0.84	-	-
Prior immunotherapy response (Responder vs. Resistant)	1.10–3.96	0.024	1.05–3.73	0.035
Consolidate radiotherapy (yes vs. no)	0.64–2.04	0.65	-	-
Performance status (0/1 vs. 2)	0.06–3.26	0.43	-	-
Primary brain metastasis (yes vs. no)	0.65–2.63	0.46	-	-
Previous radiotherapy (yes vs. no)	0.68–2.13	0.52	-	-

### Subgroup analysis

**Fig. 4:**
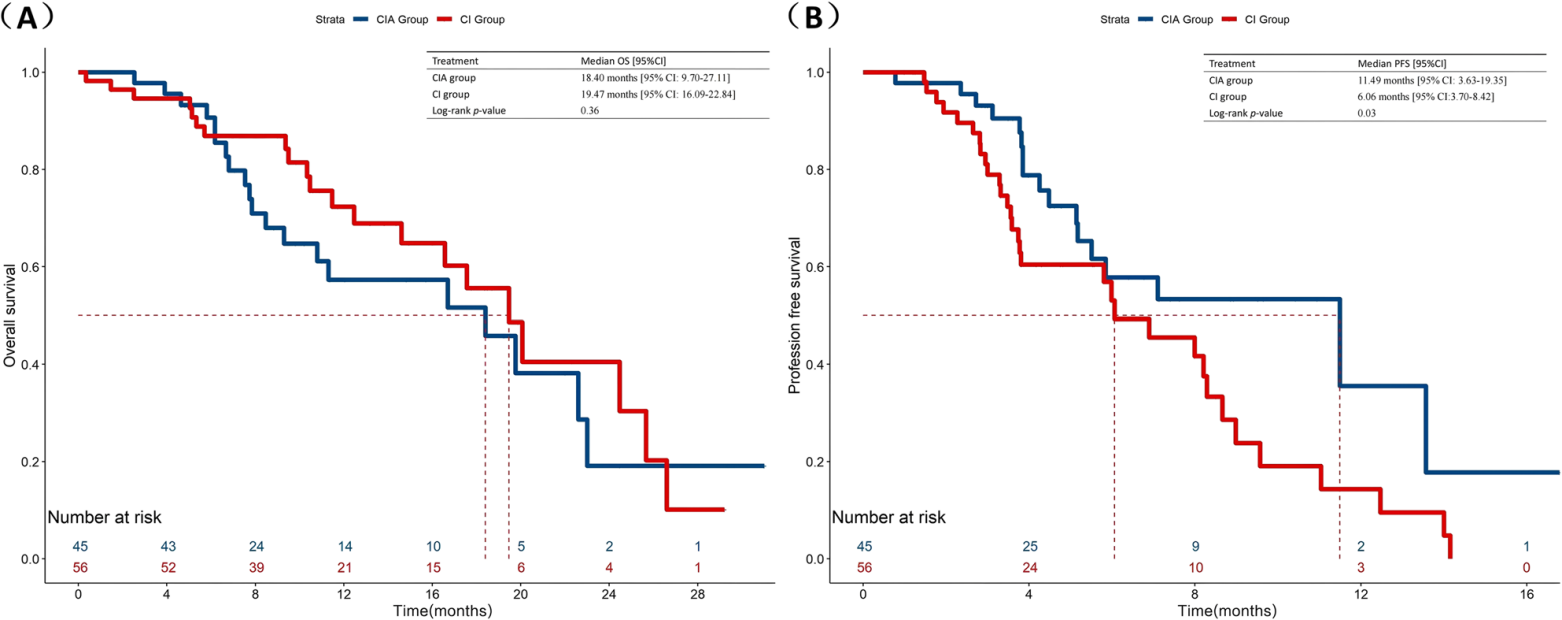
Comparison of OS and PFS in CIA subgroup based on prior immunotherapy response between 1 L-Responder Group and 1 L-Resistant Group. **A** Comparison of OS. **B** Comparison of PFS. 1 L-Responder Group, 1 L-PFS ≥ 6 months; 1 L-Resistant Group, 1 L-PFS < 6 months

**Fig. 5:**
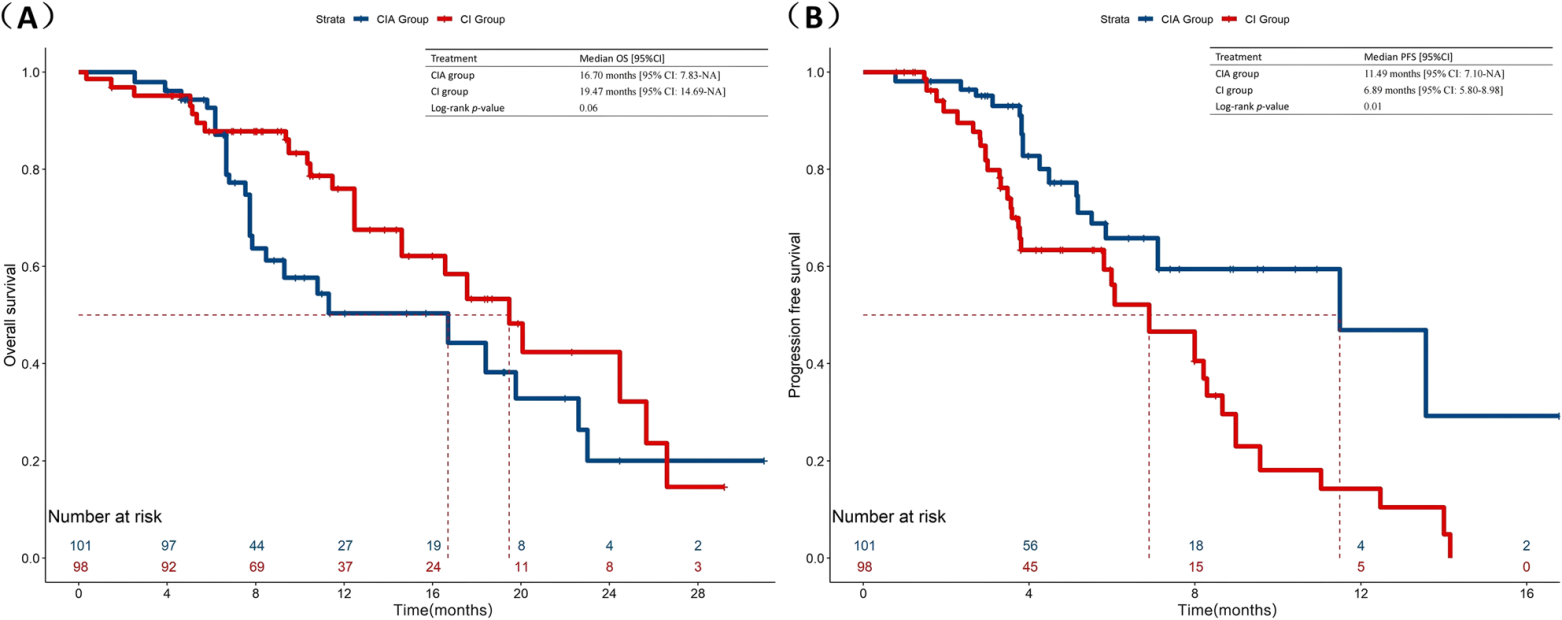
Comparison of OS and PFS in CI subgroup based on prior immunotherapy response between 1 L-Responder Group and 1 L-Resistant Group. **A** Comparison of OS. **B** Comparison of PFS. 1 L-Responder Group, 1 L-PFS ≥ 6 months; 1 L-Resistant Group, 1 L-PFS < 6 months

To further investigate the differences in survival outcomes associated with varying immune responses to prior immunotherapy among populations undergoing different immunotherapy rechallenge combination therapy modalities, we conducted a subgroup analysis. Among those who received anti-angiogenesis as part of the immunotherapy rechallenge, the median OS was observed to be 18.40 months [95% CI: 13.88–22.93] for the responder group (1 L-PFS ≥ 6 months), in contrast to 11.3 months [95% CI: 6.89–15.71] for the resistant group (1 L-PFS < 6 months) (*p* = 0.46). A similar trend was observed in the CI subgroup, the median OS was 20.07 months [95% CI:16.40-23.64] for the responder group and 14.60 months [95% CI:11.45–17.75] for the resistant group, respectively (*p* = 0.36). Notably, the survival benefit was more pronounced in the short-term outcomes of second-line treatment. Responders showed a numerically longer median PFS compared to resistant patients in the CI cohort (8.20 vs. 3.55 months, *p* = 0.02). Though in CIA subgroup these differences did not reach statistical significance (11.49 vs. 5.84 months, *p* = 0.36), more information is presented in Figs. 4 and 5.

## Discussion

Current knowledge regarding immunotherapy rechallenge is limited and primarily based on a small number of studies and exploratory data analyses. Several retrospective studies have suggested that immunotherapy rechallenge may provide clinical benefits for some patients with advanced NSCLC [19–22]. Our research findings further substantiate the potential survival benefits of immunotherapy rechallenge in patients with advanced NSCLC who have developed immunotherapy resistance, thereby contributing additional theoretical evidence to this emerging field. In this retrospective study, we provided efficacy analysis of subsequent therapy after first-line failure for advanced NSCLC patients. We indicated that immunotherapy rechallenge beyond 1 L immunotherapy progression showed survival benefits, with a median OS of 19.47 months and median PFS of 7.10 months. Based on the 2 L treatment modality, we compared the efficacy of the two combination therapies as second-line treatment. The potential combination of anti-angiogenesis agents with chemo-immunotherapy as a 2 L treatment may yield a better short-term response, evidenced by a median PFS of 11.49 months, in comparison to chemo-immunotherapy alone. However, this combination did not demonstrate significant OS benefits. After IPTW, the difference in the baseline clinical characteristics of the CIA and CI groups was controlled within a certain range. Regarding short-term efficacy, the immunotherapy rechallenge plus anti-angiogenesis therapy could still bring PFS benefits. Moreover, we classified patients based on their responses to prior immunotherapy. For those patients who responded to 1 L immunotherapy, immunotherapy beyond progression strategy showed a more favorable trend in short-term survival compared to those who exhibited resistance.

Despite immunotherapy has brought about notable advancements in survival outcomes, research has indicated that a portion of NSCLC patients might face relapse post-immunotherapy, with a considerable percentage exhibiting primary resistance [23, 24]. Immunotherapy rechallenge beyond progression has been reported as a potentially effective strategy for patients with advanced melanoma and renal cell carcinoma [6–8]. Chiarion et al. examined 855 patients with advanced melanoma, finding that the median OS of the immunotherapy rechallenge group was significantly longer than that of the control group (21 months vs. 13 months, *p* < 0.0001) [6]. Additionally, a study involving 69 patients with metastatic renal cell carcinoma indicated that switching to a second immunotherapy challenge may confer survival benefits [25]. Our study corroborates similar conclusions, demonstrating that the immunotherapy rechallenge strategies employed among the enrolled patients yielded excellent survival outcomes, with a median OS of 19.47 months and median PFS of 7.10 months. In contrast, the current CSCO guidelines recommend docetaxel monotherapy as a second-line treatment, which has an approximate median OS of 13 months and a median PFS of 3 months [26].

The combinations of immunotherapy and anti-angiogenic agents significantly improved the clinical survival in Chinese patients with unresectable hepatocellular carcinoma in the phase III IMbrave150 study. In addition, thus combination strategies have also been explored as promising treatments following the progression of immunotherapy [8, 27]. Lee’s study indicated that bevacizumab plus atezolizumab therapy showed promising anti-tumor activity in patients with advanced NSCLC progressing from 1 L platinum-containing chemotherapy [28]. Anti-angiogenesis normalizes tumor vasculature and modulates the microenvironment by increasing lymphocyte infiltration, reducing Tregs and MDSCs proliferation [15]. The synergistic interaction between immune reactivation and tumor vascular normalization mutually reinforces each other, ultimately facilitating immune-mediated tumor eradication. In our study, incorporating anti-angiogenic drugs into the regimen of immunotherapy rechallenge significantly prolonged the PFS, reaching 11.49 months. After IPTW analysis, the differences between the two groups were still significant. However, we also observed that the application of anti-angiogenic drugs did not significantly increase short-term efficacy indicators such as ORR and DCR. This might be related to the fact that patients in the terminal stage have a large burden of systemic tumor lesions, and the terminal stage of tumors is characterized by disordered angiogenesis, hypoxic microenvironment, and drug resistance, which makes it difficult for anti-angiogenic drugs to achieve significant tumor shrinkage effects. Based on the results of this study and the latest review of previous research, combining anti-angiogenic drugs on the basis of immunotherapy rechallenge may be a promising treatment strategy for those patients with advanced NSCLC progressing 1 L immunotherapy.

To more clearly define the patient population that benefits from immunotherapy rechallenge, we retrospectively analyzed clinical characteristics associated with survival benefits. Multivariate analysis revealed that patients who had a favorable response to first-line immunotherapy experienced a significantly prolonged PFS when undergoing immune rechallenge. This finding is innovative compared to previous studies on immunotherapy rechallenge. For example, the research by Vauchier and Niki primarily explored the relationship between initial immunotherapy treatment response and OS, with limited focus on the impact of initial immunotherapy treatment response on short-term efficacy [20, 29]. In our study, patients were classified into responder and resistant groups based on whether their PFS after first-line immunotherapy was less than 6 months. Our results indicated that responders, compared to resistant patients, demonstrated a significant therapeutic advantage in preventing tumor progression with immunotherapy rechallenge. Therefore, this study further enriches the theoretical basis for immunotherapy rechallenge strategies in terms of short-term efficacy benefits and emphasizes the necessity of applying immunotherapy rechallenge strategies to patients who show a good initial response to first-line immunotherapy.

As a retrospective analysis, our study has several limitations. First, this is a single-center study, and the strategy of immunotherapy rechallenge after prior immunotherapy use has not been widely endorsed on a national scale, which poses significant challenges to cohort expansion. This limitation may affect the statistical power and introduce potential bias in data interpretation, particularly in subgroup analyses of survival outcomes. Subsequently, as the patient cohort expands, we plan to increase the sample size to further validate and refine our findings. Finally, the baseline characteristics accessible in our retrospective study were constrained. For instance, crucial factors that could response to immunotherapy, such as PD-L1 expression levels or Tumor Mutation Burden (TMB), were unavailable for most of the patients enrolled in our study. Thus, our findings warrant more extensive, prospective cohort studies.

## Conclusions

Immunotherapy rechallenge provides survival benefits, and the combination of anti-angiogenic drugs with immunotherapy rechallenge may represent an optimal strategy for advanced NSCLC patients who have progressed on immunotherapy, particularly for those demonstrating favorable responses.

## Supplementary Information

Supplementary Material 1.

## Data Availability

Data generated and/or analyzed during the current study are available from the authors upon request.
