# Supplementary material for: The efficacy analysis of immunotherapy rechallenge after progression from first-line chemo-immunotherapy in advanced non-small cell lung cancer
Source: BMC Immunol. 2026 Jan 27;27:17. doi: 10.1186/s12865-026-00800-4 (PMC12918556; doi:10.1186/s12865-026-00800-4)
Supplement: Supplementary file 1 — Supplementary Material 1. [file 12865_2026_800_MOESM1_ESM.docx]

| Table S1 Summary of treatment plans | |
| --- | --- |
| The first-line regimens | Sintilimab + Pemetrexed + Carboplatin, Sintilimab + Gemcitabine + Nedaplatin, Pembrolizumab + Pemetrexed + Carboplatin, Pembrolizumab + Albumin-bound Paclitaxel + Carboplatin, Tislelizumab + Albumin-bound Paclitaxel + Cisplatin, Tislelizumab + Paclitaxel + Carboplatin, Toripalimab + Etoposide + Cisplatin. |
| The second-line regimens | Carboplatin, Camrelizumab + Albumin-bound Paclitaxel, Camrelizumab + Pemetrexed + Bevacizumab, Sintilimab + Pemetrexed + Carboplatin, Tislelizumab + Pemetrexed + Carboplatin, Tislelizumab + Endostar. |
